# Comparative Genomics Analysis of Plasmid pPV989-94 from a Clinical Isolate of *Pantoea vagans* PV989

**DOI:** 10.1155/2018/1242819

**Published:** 2018-05-10

**Authors:** Lei Xu, Min Yin, Tingyuan Zhu, Yabo Liu, Yuanyuan Ying, Junwan Lu, Chaoqing Lin, Jianchao Ying, Teng Xu, Liyan Ni, Qiyu Bao, Shunfei Lu

**Affiliations:** ^1^School of Laboratory Medicine and Life Science/Institute of Biomedical Informatics, Wenzhou Medical University, Wenzhou 325035, China; ^2^School of Medicine, Lishui College, Lishui 323000, China; ^3^The Second Affiliated Hospital, Wenzhou Medical University, Wenzhou 325000, China

## Abstract

*Pantoea vagans*, a gram-negative bacterium from the genus *Pantoea* and family *Enterobacteriaceae*, is present in various natural environments and considered to be plant endophytes. We isolated the *Pantoea vagans* PV989 strain from the clinic and sequenced its whole genome. Besides a chromosome DNA molecule, it also harboured three large plasmids. A comparative genomics analysis was performed for the smallest plasmid, pPV989-94. It can be divided into four regions, including three conservative regions related to replication (R1), transfer conjugation (R2), and transfer leading (R3), and one variable region (R4). Further analysis showed that pPV989-94 is most similar to plasmids LA637P2 and pEA68 of *Erwinia amylovora* strains isolated from fruit trees. These three plasmids share three conservative regions (R1, R2, and R3). Interestingly, a fragment (R4′) in R4, mediated by phage integrase and phage integrase family site-specific recombinase and encoding 9 genes related to glycometabolism, resistance, and DNA repair, was unique in pPV989-94. Homologues of R4′ were found in other plasmids or chromosomes, suggesting that horizontal gene transfer (HGT) occurred among different bacteria of various species or genera. The acquired functional genes may play important roles in the adaptation of bacteria to different hosts or environmental conditions.

## 1. Introduction

The genus *Pantoea* includes approximately 20 phenotypically similar yellow-pigmented and rod-shaped species in plants, humans, and the natural environment [1]. It is closely related to the *Erwinia* and *Tatumella* genera, which are grouped as sisters [2]. For classification and identification of *Pantoea* species, the four housekeeping genes, *gyrB*, *rpoB*, *atpD*, and *infB*, are reliable genetic markers [3]. *Pantoea vagans* belongs to the genus *Pantoea* and is separated from *Pantoea agglomerans* based on borderline DNA-DNA relatedness, 16S rRNA, multilocus sequence analysis (MLSA), and several biochemical characteristics [3]. A portion of *Pantoea vagans* strains have been isolated and screened from natural resources such as a Malaysian waterfall and French soil and sea water. In addition, the bacterium 848_PVAG has been isolated from a patient in the intensive care unit of a tertiary care hospital [4], while the majority of strains are epiphytes attached to a broad range of plants. The typical strain of *Pantoea vagans* is strain C9-1 from Michigan apples. It has been registered as BlightBan C9-1 (NuFarm Americas, Burr Ridge, IL) and works as causal biocontrol for fire blight disease in pome fruit caused by the enterobacterium *Erwinia amylovora* [5, 6].

Canonically horizontal gene transfer (HGT) is dependent on mobile genetic elements (MGEs) that include plasmids, transposons, bacteriophages, genomic islands, and integrative conjugative elements (ICEs). Some MGEs such as phage integrases may be inserted in strategic locations and serve to mobilize certain genes [7]. Phage integrases are classified into two evolutionarily and mechanistically different families of site-specific recombinases, the tyrosine recombinases and the serine recombinases, which are differentiated by the amino acid residue that forms a covalent protein-DNA linkage in the reaction intermediate [8]. The bacterial attachment site *attB* and phage attachment site *attP* are recognized by phage integrases during integration and the excision reaction, and the mobile genetic element emerges as integrated prophages flanked by two hybrid sites, *attL* and *attR* [9]. The site-specificity and efficiency of phage integrases are essential for the HGT of genetic information and the control of gene expression.

To date, 22 species from the genus *Pantoea* have been sequenced, of which 7 *Pantoea* species have complete genomes. Genome sequencing showed that two strains of *Pantoea agglomerans* and one strain of *Pantoea vagans* harbored three plasmids, whereas one strain of *Pantoea rwandensis* was free of plasmids. In this study, we isolated a clinical strain designated *Pantoea vagans* PV989. We sequenced the complete genome of the strain and analyzed the structural components of the plasmid pPV989-94. In addition, we also performed extensive comparative analysis among the plasmid pPV989-94 and its close relatives to explore their origin, evolution, and distribution.

## 2. Results and Discussion

### 2.1. Identification and Characterization of *Pantoea vagans* PV989

*Pantoea vagans* PV989 was isolated from the blood of a high-risk newborn infant girl with bacteremia at the Central Hospital of Lishui City, China. We used single molecule real time (SMRT) technology to determine the complete genome sequence of *Pantoea vagans* PV989. The whole genome comparative analysis combined with 16S ribosomal RNA gene homology analysis showed that the most closely related species were two *Pantoea vagans* strains (*P. vagans* C9-1, CP002206 and *P. vagans* LJ15, KM40861), and the most similar genome belongs to *Pantoea vagans* C9-1. It has been reported that the multilocus sequence analysis (MLSA) of housekeeping genes is a useful approach for rapid classification and identification of *Pantoea* strains with high phenotypic similarity and is more reliable than 16S rRNA sequences [10]. Four housekeeping genes, namely, *gyrB*, *rpoB*, *atpD*, and *infB*, from the *Pantoea vagans* PV989 genome were further evaluated and showed optimum matching to those in *P. vagans* C9-1 (identity 100%, 99%, 99%, and 99%, resp.). We finally grouped the strain into the species *Pantoea vagans* and named it *Pantoea vagans* PV989.

### 2.2. General Features of the Genome of *Pantoea vagans* PV989

**Figure 1:**
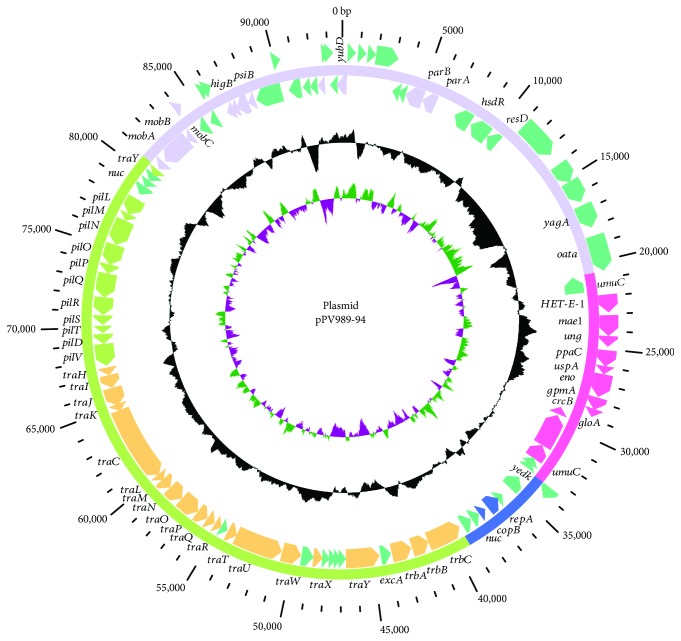
The circular map of plasmid pPV989-94. (1) The circle on the outside shows positions in bp. (2) The four regions (replication, R1; transfer conjugation, R2; transfer leading, R3; and the variable region, R4) in the circle are in blue, green, purple, and pink, respectively. Arrows display ORFs on the lagging strand (outwards) or leading strand (inwards), with ORFs involved in replication in blue, conjugation in light yellow, pilus formation in light green, transfer leading in light purple, MGEs in pink, and others in blue-green. (3) The G + C content with values more than 50% are directed outward; otherwise, inward; (4) GC skew (G − C/G + C) with the positive values directed outward are in green, and the negative values directed inward are in purple.

The genome of *Pantoea vagans* PV989 consists of a chromosome and three large plasmids. The chromosome is 4,071,006 bp in length and encodes 3648 ORFs. The largest plasmid, named pPV989-508, is 507,680 bp in length and harbors 492 ORFs. The second largest plasmid, pPV989-167, is 166,637 bp in size and carries 152 ORFs, and the smallest plasmid, pPV989-94, is 93,831 bp in length and has 101 ORFs (Table 1). Based on the sequence structures and the functions of its ORFs, plasmid pPV989-94 can be roughly divided into four parts (Figure 1). The first region (R1, 7 ORFs from ORF41 to ORF48) relates to plasmid replication and consists of *repA*, *copB*, *yedk*, and *nuc* and 3 unknown ORFs. The second region (R2, 42 ORFs from ORF49 to ORF113), next to R1, is mainly composed of three operons (3 *trb*, 18 *tra*, and 11 *pil* genes) related to conjugal transfer, the genes *excA*, *ymoA*, *ppg1*, and *nuc*, lipoprotein, and five ORFs whose functions are unknown. The third region (R3, 38 ORFs, from ORF114 to ORF19) is defined as the transfer leading region and includes ORFs for the mobilization system (*mobA*, *mobB*, and *mobC*) and plasmid SOS inhibition (*psiA*, *psiB*). The fourth region (R4, 14 ORFs from ORF20 to ORF40) mainly contains a mobile genetic element. It includes a fragment (named R4′) inserted into the *umuC* gene, which is characterized by a phage integrase (ORF38) and a phage integrase family site-specific recombinase (ORF37) and encodes 9 genes from different origins with various functions.

### 2.3. Structural Analysis of Plasmid pPV989-94

**Figure 2:**
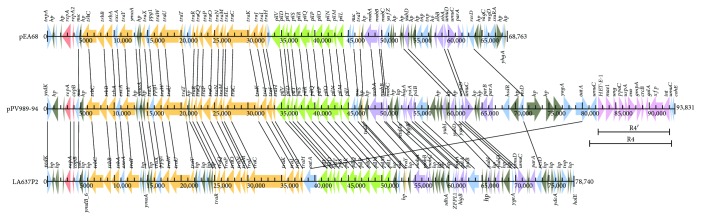
Comparison of the genome structure of the plasmids pPV989-94, LA637P2, and pEA68. The homologous genes (AA identity > 50%) are linked with lines.

To explore the origin of the pPV989-94 plasmid, two plasmids with the highest sequence similarities with pPV989-94 were identified. Both are plasmids from *Erwinia amylovora* strains (LA637P2, HG793099.1, a 78 kb plasmid from *Erwinia amylovora* LA637 and pEA68, and HG813238.1, a 68 kb plasmid from *Erwinia amylovora* 692). Comparative genomics analysis showed that they had 46.8 kb in common, including the R1, R2, and R3 regions in pPV989-94 (Figure 2). They all harbor homologous genes including 3 *trb*s and 18 *tra*s encoding a type IV F conjugative transfer system, 11 *pil*s encoding a type IV pilus system, *mobABC* encoding a mobilization protein, and *umuCD* for DNA error-prone repair. Plasmid pPV989-94 is approximately 12 kb larger than LA637P2, which mainly consists of the R4 region, and 22 kb larger than pEA68, which covers part of the R3 and R4 regions. Alignments of the three plasmids reveal synteny over long stretches of the plasmid genome. Despite the high similarity of the backbone of the plasmids, the origins of the host bacteria are totally different. The pPV989-94 plasmid carried by the strain *P. vagans* PV989 is a clinical isolate from southern China, while pEA68 and LA637P2 harbored by *Erwinia amylovora* strains are isolated from two continents, the former from *Sorbus* in Poland with fire blight symptoms and the latter from apple orchards in Mexico [11, 12].

In addition, each of the plasmids possesses its own variable regions. A toxin-antitoxin system (*vagCD*), an atypical plasmid stabilization system (*stbAB*) and several transposases (*tnp*s) are present in pEA68. An IncFIIa-type *repA* system in pEA68 shows low identity with IncFII *repA* in LA637P2 or pPV989-94. IncF plasmids are subject to the host restriction of the Enterobacteriaceae family and possess strong intracellular adaptation [13]. Genes encoding various small proteins and hypothetical proteins are divergent in all three plasmids. Moreover, the phage integrase-mediated fragment (R4′) inserted into *umuC* is exclusively in pPV989-94. The integration confers a level of specialization and contributes to interstrain genetic variability and genome diversity.

### 2.4. Comparative Genomics Analysis of the Mobile Genetic Element in R4

**Figure 3:**
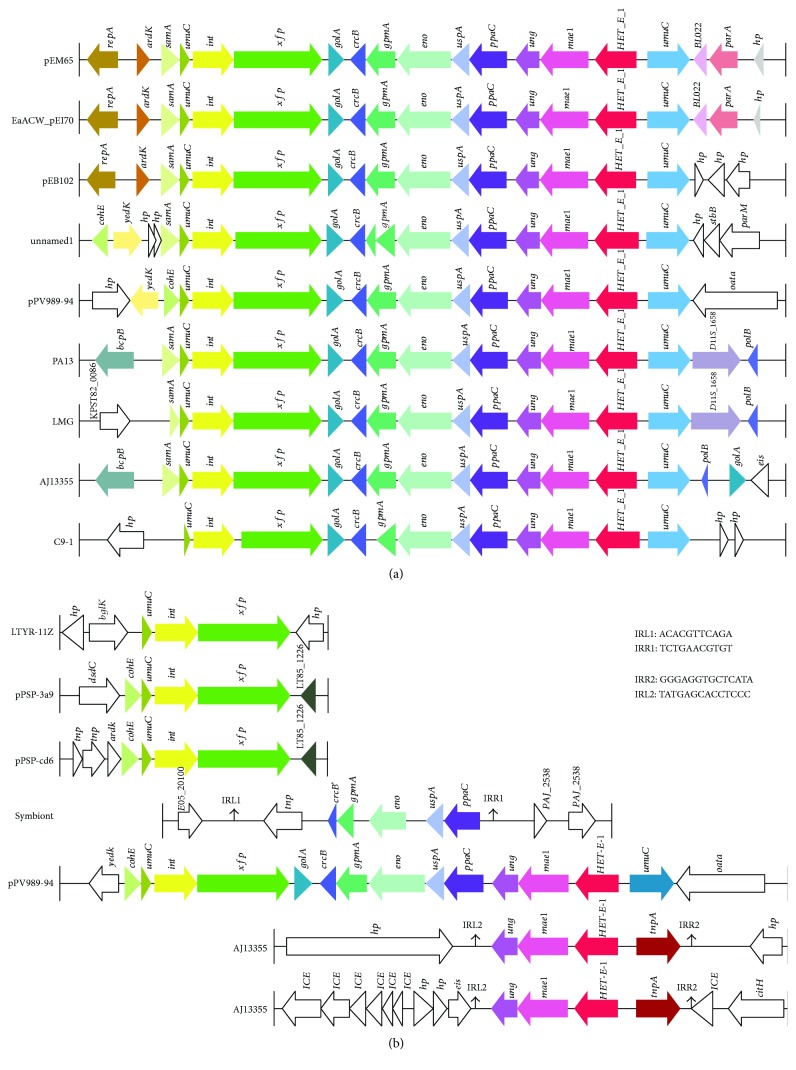
Comparative genomic analysis of the structure of R4′ and its homologous fragments. (a) Comparison of the structure of R4′ with the homologous fragments of the entire R4′. (b) Comparison of the structure of R4′ with homeotic partial R4′ fragments. Homologous genes are in the same colors, whereas the white arrows mean the nonhomologous genes.

The variable regions make the plasmids different in size and genomic structure. Fragment R4′ in pPV989-94 encodes 11 genes that span from the phage integrase (ORF38) to PQQ-dependent catabolism-associated beta-propeller protein (*HET-E-1*, ORF22) in R4 and is 10.7 kb in length (Figure 2). All of the homologous genes of fragment R4′ were found in eight bacterial chromosomes or plasmids, including the chromosomes of *Pantoea vagans* C9-1 (CP002206.1), *Pantoea ananatis* PA13 (CP003085.1), *Pantoea ananatis* LMG 5342 (HE617160.1), and *Pantoea ananatis* AJ13355 (AP012032.2) as well as plasmid unnamed1 from *Pantoea agglomerans* FDAARGOS_160 (CP014126.1), plasmid pEB102 from *Erwinia billingiae* Eb661 (FP236826.1), plasmid pEM65 from *Erwinia amylovora* (JQ292796.1), and plasmid EaACW_pEI70 from *Erwinia amylovora* CW56400 (CP002951.1) (Table 2 and Figure 3(a)). These bacteria were mainly isolated from plants such as rice or clinical patient wounds polluted by plant materials from several countries (Table 2).

Interestingly, among eight R4′ fragments, the sequences in the plasmids pEM65 (*E. amylovora*), EaACW_pEI70 (*E. amylovora*), pEB102 (*E. billingiae*), and unnamed1 (*P. agglomerans*) are almost identical, with at least 99% identity to each other at the nucleic acid (NA) level. Similarly, the sequence from the *P. vagans* C9-1 chromosome also has 99% NA identity with the sequences from those four plasmids. For the chromosome sequences of R4′ homologous regions from *P. ananatis* PA13, *P. ananatis* LMG 5342, and *P. ananatis* AJ13355, the NA identities are more than 98%. Surprisingly, the R4′ sequence in plasmid pPV989-94 was closer to those on the *P. ananatis* LMG 5342 and *P. ananatis* PA13 chromosomes than it was to that on the *P. ananatis* AJ13355 chromosome and was least similar to those on the *P. vagans* C9-1 chromosome and the four plasmids (pEM65, EaACW_pEI70, pEB102, and unnamed1). These results suggest that the R4′ homologous fragments are distributed in the *Pantoea* and *Erwinia* genera and that the HGT of this fragment occurred between chromosomes and plasmids in different species or genera.

Unexpectedly, homeotic partial R4′ fragments were also found (Figure 3(b)). In the chromosome of AJ13355, besides one entire R4′ homologous fragment, two copies of partial R4′ fragments of three genes (*HET-E-1*, *mae1*, and *ung*) were also present in the chromosome. Structural analysis showed that both copies were mediated by IS110 family transposases (*tnpA*s) with 14 bp perfect inverted repeats (IR1) lying upstream of *tnpA*s and downstream of *ung*s, which are typical transposons. Moreover, each gene of the two transposons shows a high degree of similarity with that in the whole R4′ fragments of AJ13355 and pPV989-94. Furthermore, a partial R4′ fragment encoding the genes *crcB*, *gpmA*, *eno*, *uspA*, and *ppaC* mediated by an IS911 family transposase appears in *Plautia stali* symbiont DNA (AP012551.1), which is the symbiotic bacterium of the stink bug *Plautia stali*. There are two 11 bp perfect inverted repeats flanking *tnpA* and *ppaC* (IR2). However, other partial fragments (CP019706.1, CP010326.1, and CP009871.1) only containing phage integrases and recombinases have also been identified, indicating that the entire R4′ has been further fragmented by MGEs to spread among strains of the different species or genera.

### 2.5. Functional Analysis of the Genes in the R4′ Fragment

There were 9 ORFs predicted in the R4′ fragment other than the phage integrase and the site-specific recombinase genes. The functional annotations revealed that they were related to glycometabolism (*gloA*, g*pmA*, *eno*, *ppaC*, *mae1*, and *HET-E-1*), resistance (*crcB*, *uspA*) and DNA repair (*ung*).

#### 2.5.1. Glycometabolism-Associated Genes and Their Functions

*gloA* encodes glyoxalase I, an (R)-S-lactoylglutathione methylglyoxal lyase (isomerizing) activated by Zn(II) or Ni(II) ions, and is 411 bp (136 amino acids) in length. It has been identified in bacteria, fungi, protists, plants, and animals but not in the protozoans *Entamoeba histolytica*, *Giardia lamblia*, and *Trypanosoma brucei* [14]. Catalysis of the glyoxalase I is the first step of the typical glyoxalase pathway wherein glyoxalase I catalyzes toxic methylglyoxal (MG) and generates innocuous S-D-lactoylglutathione, which then enters the mitochondria from the cytosol with glutathione (GSH) [15]. MG is a noxious electrophilic product of dihydroxyacetone phosphate (DHAP), which is one of the two products of the breakdown of fructose 1,6-bisphosphate in the glycolysis metabolic pathway. The GSH-dependent glyoxalase system suppresses *α*-oxoaldehyde-mediated glycation, helps maintain low intracellular reactive MG concentrations, and protects cells from irreversible damage in glycation [16]. It plays an irreplaceable role in antiglycation defense and protects bacteria from poisonous MG for survival.

*gpmA* and *eno* encode phosphoglycerate mutase and enolase, respectively, which have been known to participate in substrate level phosphorylation as glycolytic enzymes in carbon metabolism. The sequence of enolase is highly conserved from archaebacteria to mammals [17]. It has been found on the cell surfaces or in the culture supernatant of bacteria [18]. The *gpmA* and *eno* genes in the *E. amylovora* ACW56400 plasmid pEI70 have been reported to increase growth rates in minimal medium with sucrose, thus improving competence and increasing pathogen aggressiveness [19].

*ppaC* encodes inorganic pyrophosphatase (PPase) with the DHHA2 domain (IPR004097) and pyrophosphatase activity (GO: 0016462). It is well known that PPase can irreversibly hydrolyze one molecule of inorganic pyrophosphate (PPi) that comes from the processes of photophosphorylation, oxidative phosphorylation, and glycolysis into two inorganic phosphates (Pi). It is a highly exergonic reaction driving biosynthetic reactions, such as NA synthesis and lipid synthesis and degradation.

Enolase and PPase are highly sensitive to fluoride, and most organisms have equipment such as phosphoryl-transfer enzymes either to sequester or to export fluoride [20, 21]. Fluoride ions present at millimolar concentrations in bacterial culture media inhibit cell growth. The *crcB*-encoded CrcB protein is a “double-barreled” anion membrane channel and functions as a fluoride ion transporter that selectively exports many toxic fluoride ions [22]. The expression of the CrcB protein is controlled by fluoride-sensing messenger RNAs named fluoride riboswitches. The CrcB protein augments the activity of CspE to increase camphor resistance beyond the action of *crcB* alone, which protects the chromosome from decondensation by camphor [23]. It seems that CrcB significantly reduces fluoride concentration and chromosome decondensation to alleviate toxicity and hazards in the environment.

#### 2.5.2. Resistance-Associated Genes and Their Functions

Universal stress protein A (UspA) expressed monocistronically in living systems is an autophosphorylating serine and threonine phosphoprotein and acts as a phosphate donor when it utilizes GTP or ATP [24]. Except for ppGpp, the *uspA* promoter is affected by the activities of the glycolytic/gluconeogenic pathways and is induced before the carbon source is depleted from the medium to generate adaptation to energy deficiency. The superinduction of *uspA* is elicited by the accumulation of fructose-6-phosphate (F-6-P), whereas a decrease in F-6-P has the opposite effect [25]. Furthermore, UspA is important in the defense against stress conditions, such as low oxygen, extreme temperatures, pH changes, nutrient starvation, oxidative stress, and DNA damaging agents, for avoiding cellular growth arrest [26].

The C4-dicarboxylate transporter is encoded by *mae1*, which contains the tellurite resistance protein TehA/malic acid transport protein domain (IPR011552) of the voltage-dependent anion channel family. TehA was identified as a transmembrane protein from *Escherichia coli* that was resistant to tellurite, tetraphenylarsonium Cl, ethidium bromide, crystal violet, and proflavin [27], and the malic acid transport phenotype increases L-malic acid uptake by competing against stearic dicarboxylic acids in *Schizosaccharomyces pombe* [28].

*HET-E-1* was associated with the PQQ-dependent catabolism-associated beta-propeller protein (HET-E-1) containing alterable repeats of the YVTN family beta-propeller. The members of this family are often involved in pyrroloquinoline quinone-dependent (PQQ-dependent) alcohol catabolism. The functions of PQQ alcohol dehydrogenase are redundant for adaptation to changing environments. Thus, the PQQ-dependent oxidation system plays a significant role in efficient growth with various volatile alcohols [29].

#### 2.5.3. DNA Repair-Associated Genes and Their Functions

Uracil-DNA glycosylase (UNG) has the isogenous domain uracil-DNA glycosylase family 4 (IPR005122 and IPR005273), which hydrolyzes the N-glycosidic bond of deoxyuridine in DNA to initiate DNA repair after lesioning in viruses, bacteria, archaea, yeast, mice, and humans. The structure of UNG is a specific uracil-binding pocket located in a DNA-binding groove that binds to DNA with appreciable affinity [30]. UNG is a monofunctional glycosylase and removes the uracil that results from spontaneous deamination of cytosine or the incorporation of dUMP specifically during DNA synthesis [31]. UNGs are classified into six families by their substrate specificity, and the IV UNG family interacts with one of the heterotrimeric proliferating cell nuclear antigen (PCNA) subunits, PCNA3, in *Sulfolobus solfataricus* [31]. UNGs were successively discovered in the hyperthermophilic bacterium *Termotoga maritima* as well as in archaea and only acts on double-stranded DNA containing U : G mispairs and single-stranded DNA containing U : A base pairs or U bases [32]. The base excision function of UNG maintains genetic stability and genomic integrity, particularly after UV radiation or exposure to environmental mutagens.

## 3. Conclusion

*Pantoea vagans* PV989 is a clinical isolate, and it harbors three large plasmids. Comparative genomics analysis demonstrated that two plasmids, LA637P2 and pEA68, from *Erwinia amylovora* strains isolated from fruit trees in Europe and North America, respectively, showed the highest sequence similarity with the smallest plasmid pPV989-94 in *P. vagans* PV989. The three plasmids shared the conservative backbone sequences consisting of the genes relating to replication, the type-IV F conjugative transfer system, and transfer leading. However, plasmid pPV989-94 contained a variable region that was a mobile genetic element mediated by the phage integrase and site-specific recombinase, and the region carried genes related to glycometabolism, resistance, and DNA repair. The acquired functional foreign genes guarantee the bacterium's ability to adapt to environments, including the human body, and certainly pose a threat to human health.

## 4. Materials and Methods

### 4.1. Bacterial Strain and Plasmids

The host strain *P. vagans* PV989 that contains three plasmids was isolated from the Central Hospital of Lishui City, China. Species identification was conducted using a bioMérieux VITEK® 2 Compact instrument. Further verification was performed using homologous comparisons of seven 16S rRNA gene sequences, the whole genome sequence, and housekeeping genes with bacteria of the same genera in the NCBI database through blastn and blastp [33]. Seven 16S rRNA sequences were found by the online tool RNAmmer (http://www.cbs.dtu.dk/services/RNAmmer/) [34]. The plasmid pPV989-94 is the smallest of the three plasmids of *P. vagans* PV989.

### 4.2. Sequencing, Assembly, and Bioinformatics Analysis

The whole genome DNA of *P. vagans* PV989-9 was extracted by the alkaline lysis method [35]. The draft genome sequence of *P. vagans* PV989 was obtained using a PacBio RS instrument and was then assembled by Canu v1.2 [36]. Two FASTQ sequence files were generated using the Illumina HiSeq 2500 platform to control the assembly quality and to correct possible misidentified bases. Single nucleotide polymorphism (SNP) calling was performed by bwa0.7.13, samtools1.3, and GenomeAnalysisTK2.3.9 [37, 38]. Potential open reading frames (ORFs) were predicted using Glimmer3.02 with default parameters [39]. The annotations of ORFs were determined by using tblastx compared with the NCBI nonredundant protein database [33]. The comparisons of nucleotide sequences and amino acid sequences were performed by blastn and blastp, respectively [33]. The map of the plasmid with GC content and GC skew was drawn with the online CGView Server (http://stothard.afns.ualberta.ca/cgview_server/) and local GView 1.7 with visual interface [40]. The plasmid and chromosome genome sequences used in this study were downloaded from the NCBI database (http://www.ncbi.nlm.nih.gov). Family classification and domain prediction of the MGEs were verified by comparison with the InterPro database [41]. Other bioinformatics tools were written using Perl and BioPerl (http://www.perl.org/).

### 4.3. Accession Number

The complete nucleotide sequences of the chromosome and plasmids have been submitted to a NCBI database and the accession numbers of the chromosome, pPV989-508, pPV989-167, and pPV989-94 are CP028349.1, CP028350.1, CP028351.1, and CP028352.1, respectively.

## Figures and Tables

**Table 1:** The general features of the genome of *Pantoea vagans* PV989.

	Chromosome	pPV989-508	pPV989-167	pPV989-94

Size (bp)	4,071,006	507,680	166,637	93,831
% GC	55.59	54.00	53.02	54.39
Total open reading frames (ORFs)	3648	492	152	101
tRNA	79	0	0	0
rRNA	22	0	0	0

**Table 2:** Complete and partial R4′ homologous fragments and their origins.

Strain	Genome	Host	Origin	Reference

C9-1	Chromosome	Apple	Michigan	Complete [6]
PA13	Chromosome	Diseased rice	Korea	Complete [42]
LMG 5342	Chromosome	Human wound	Georgia	Complete [43]
AJ13355	Chromosome	Soil	—	Complete, partial [44]
FDAARGOS_160	Plasmid unnamed1	Human wound	USA: DC	Complete
Eb661	Plasmid pEB102	Plants	—	Complete [45]
CFBP7517	Plasmid pEM65	Pear	Middle Atlas, Morocco	Complete [46]
ACW56400	Plasmid EaACW_pEI70	Pear	Switzerland	Complete [19]
PV989	Plasmid pPV989-94	Human blood	Lishui, China	Complete, this work
*Plautia stali* symbiont	Chromosome	*Plautia stali*	Tsukuba, Japan	Partial [47]
LTYR-11Z	Chromosome	*Alhagi sparsifolia* Shap.	Desert, Xinjiang, China	Partial
PSNIH1	Plasmid pPSP-3a9	Shelf	USA	Partial [48]
PSNIH2	Plasmid pPSP-cd6	Hand rail	USA	Partial [48]
